# Rural–urban and geographical differences in prognosis of atrial fibrillation in Finland: a nationwide cohort study

**DOI:** 10.1177/14034948231189918

**Published:** 2023-08-12

**Authors:** Konsta Teppo, K.E. Juhani Airaksinen, Olli Halminen, Jussi Jaakkola, Miika Linna, Jari Haukka, Jukka Putaala, Pirjo Mustonen, Janne Kinnunen, Juha Hartikainen, Mika Lehto

**Affiliations:** 1 Heart Centre, Turku University Hospital and University of Turku, Finland; 2 Department of Industrial Engineering and Management, Aalto University, Espoo, Finland; 3 Neurology, Helsinki University Hospital, and University of Helsinki, Finland; 4 Heart and Lung Centre, Helsinki University Hospital and University of Helsinki, Finland; 5 University of Helsinki, Finland; 6 University of Eastern Finland, Kuopio, Finland; 7 Heart Centre, Kuopio University Hospital, Kuopio, Finland; 8 Jorvi Hospital, Department of Internal Medicine, Helsinki and Uusimaa Hospital District, Espoo, Finland

**Keywords:** Atrial fibrillation, prognosis, rural, urban, geographical disparities, ischemic stroke, mortality

## Abstract

**Aims:**

Rural–urban disparities have been reported in the outcomes of cardiovascular diseases. We assessed whether rural–urban or other geographical disparities exist in the risk of ischemic stroke (IS) and death in patients with atrial fibrillation (AF) in Finland.

**Methods:**

The registry-based FinACAF cohort study covers all patients with AF from all levels of care in Finland from 2007 to 2018. Patients were divided into rural–urban categories and into hospital districts (HDs) based on their municipality of residence.

**Results:**

We identified 222,051 patients (50.1% female; mean age 72.8 years; mean follow-up 3.9 years) with new-onset AF, of whom 15,567 (7.0%) patients suffered IS and 72,565 (32.7%) died during follow-up. The crude IS rate was similar between rural and urban areas, whereas the mortality rate was lower in urban areas (incidence rate ratios (IRRs) with 95% confidence intervals (CIs) 0.97 (0.93–1.00) and 0.92 (0.91–0.93), respectively). However, after adjustments, urban residence was associated with slightly higher IS and mortality rates (IRRs with 95% CIs 1.05 (1.01–1.08) and 1.06 (1.04–1.07), respectively). The highest crude IS rate was in the East Savo HD and the lowest in Åland, whereas the highest crude mortality rate was in the Länsi-Pohja HD and the lowest in the North Ostrobothnia HD (IRRs with 95% CIs compared to Helsinki and Uusimaa HD for IS 1.46 (1.28–1.67) and 0.79 (0.62–1.01), and mortality 1.24 (1.16–1.32) and 0.97 (0.93–1.00), respectively.

**Conclusions:**

**Rural–urban differences in prognosis of AF in Finland appear minimal, whereas considerable disparities exist between HDs**.

## Introduction

Rural–urban disparities have been reported in the access, utilization, and quality of healthcare [1–4]. Additionally, several studies have observed higher all-cause mortality and worse outcomes of cardiovascular diseases in populations living in rural areas when compared to urban areas [5–11]. However, in patients with atrial fibrillation (AF), the most common cardiac arrhythmia and a major risk factor for ischemic stroke (IS), studies investigating outcome differences between rural and urban areas are limited and have shown inconsistent results [12–15]. Moreover, previous studies have reported notable geographical differences in cardiovascular health in Finland, but whether similar disparities exist in the outcomes of patients with AF is unknown [16, 17]. We assessed the hypothesis that there are rural–urban and other geographic differences in the risk of IS and death in patients with AF in Finland. We also aimed to investigate the extent to which possible differences in prognosis could be explained by differences in age structure, comorbidity, and oral anticoagulant (OAC) use. Therefore, we conducted a nationwide retrospective cohort study covering all patients diagnosed with AF in Finland between 2007 and 2018.

## Methods

### Study population

The Finnish AntiCoagulation in Atrial Fibrillation (FinACAF) Study (ClinicalTrials Identifier: NCT04645537; ENCePP Identifier: EUPAS29845) is a nationwide retrospective cohort study that includes all patients documented with AF in Finland from 2004 to 2018 [18]. Patients were identified using all available national healthcare registers (hospitalizations and outpatient specialist visits: HILMO; primary healthcare: AvoHILMO; and the National Reimbursement Register upheld by Social Insurance Institute: KELA). The inclusion criterion for the cohort was an International Classification of Diseases, Tenth Revision (ICD-10) diagnosis code of I48 (including atrial fibrillation and atrial flutter, together referred to as AF) recorded between 2004 and 2018. The exclusion criteria were permanent emigration abroad before 31 December, 2018, and age < 20 years at AF diagnosis. The current sub-study was conducted within a cohort of patients with incident AF between 2007 and 2018, which was established in previous studies of the FinACAF cohort [19–21]. Patients with missing residence data were excluded. Follow-up started from the first AF diagnosis and in the analyses on IS rate follow-up continued until IS, death or 31 December, 2018, whichever occurred first. In the mortality analyses, follow-up continued until death or 31 December, 2018. Data on baseline comorbidities were gathered from the aforementioned healthcare registers. The cohort construction process is summarized in Supplementary Figure 1, and the definitions of the baseline comorbidities are presented in Supplementary Table 1.

### Residence status

Finland is a large (338,440 km^2^) and relatively highly urbanized country with sparsely inhabited rural areas [22]. The patients of the study cohort were categorized into rural and urban groups according to Finland’s Environmental Administration’s rural–urban classification system and the patients’ municipality of residence at cohort entry [23]. In this classification, urban areas are agglomerations with more than 15,000 residents. Additionally, patients were divided into tertiles according to the degree of urbanization of their municipality of residence, acquired from Statistics Finland. The degree of urbanization refers to the proportion of people in a municipality living in localities or urban settlements [24]. Additionally, we classified patients into 21 hospital district (HD) categories based on the zip code of their residence.

### Outcomes

In patients who had not suffered IS prior to the first AF diagnosis, the IS event was considered to occur on the first date of a recorded I63 or I64 ICD-10 diagnosis code in the hospital care register after cohort entry. In patients with prior IS, the event was considered to occur on the date of the first new hospitalization with I63 or I64 ICD-10 code as the main diagnosis with at least a 90-day gap from the prior event, which had occurred before AF diagnosis. Furthermore, in the sensitivity analysis, I63 or I64 ICD-10 code as the main cause of death was also included in the IS outcome variable. Dates and causes of death were retrieved from the National Death Register maintained by Statistics Finland.

### Study ethics

The study protocol was approved by the Ethics Committee of the Medical Faculty of Helsinki University, Helsinki, Finland (nr. 15/2017), and was granted research permission from Helsinki University Hospital (HUS/46/2018). Respective permissions were obtained from the Finnish register holders (KELA 138/522/2018; THL 2101/5.05.00/2018; Population Register Centre VRK/1291/2019-3; Statistics Finland TK-53-1713-18 / u1281; and the Tax Register VH/874/07.01.03/2019). Patients’ personal identification numbers were pseudonymized, and the research group received individualized but unidentifiable data. Informed consent was waived due to the retrospective registry-nature of the study. The study conforms to the Declaration of Helsinki as revised in 2013.

### Statistical analyses

We estimated incidence rates and incidence rate ratios (IRRs) with 95% confidence intervals (CIs) for IS and death using a Poisson regression model with a Lexis-type data structure based on three timescales: follow-up time from AF diagnosis, calendar year, and age [25]. This statistical approach was chosen to account for the patients’ age increasing during the relatively long observation period between 2007 and 2018, as well as for the previously reported temporal differences in the outcome rates [19]. Adjusted IRRs were computed in three consecutive models, with the first model including age, calendar year, and sex. In the second model, the regressions were further fitted with the following baseline variables: heart failure, hypertension, diabetes, prior IS or transient ischemic attack, vascular disease, dyslipidemia, prior bleeding, alcohol use disorder, renal failure, liver cirrhosis or failure, cancer, dementia, psychiatric disorders and income level (divided in quartiles). Thereafter, in the third model, the regressions were further fitted with OAC-use during follow-up (Anatomical Therapeutic Chemical codes: B01AE08, dabigatran; B01AF01, rivaroxaban; B01AF02, apixaban; B01AF03, edoxaban; B01AA03, warfarin). OAC-use was treated as a time-varying variable and treatment was considered to start from the first OAC purchase and to continue until 120 days after the last drug purchase. The 120-day interval was chosen since, in Finland, it is possible to purchase drugs with reimbursement for a maximum of 90 days and an additional 30-day grace period was allowed to cover possible stockpiling and differences in warfarin dosing. The chi-square test, the Student’s *t*-test, and analysis of variance were used to compare baseline variables. Statistical analyses were performed with IBM SPSS Statistics software version 28.0 (SPSS, Inc., Chicago, Illinois, USA) and R version 4.0.5 (R Core Team, Vienna, Austria; https://www.R-project.org).

## Results

We identified 222,051 patients (50.1% female; mean age 72.8 years; standard deviation (SD) 13.1) with new-onset AF. The mean follow-up time was 3.9 years (SD 3.2) in the IS analyses. Patients with urban residence were younger and had lower prevalence of cardiovascular comorbidities, but higher income and -prevalence of psychiatric disorders than patients with rural residence (Table I). Patients with rural residence were more likely to initiate OAC therapy than urban residence patients (72.0% vs. 69.9%, *p* < 0.001). Overall, 15,567 (7.0%) patients suffered IS and 72,565 (32.7%) died during follow-up. The main cause of death was IS in 4,727 (2.1%) patients.

**Table I.** Baseline characteristics of the study cohort according to residence status.

	Rural–urban status		*P*-value	Urbanization degree tertiles			*P-*value
	Rural	Urban		1^st^ (lowest)	2^nd^	3^rd^ (highest)	
	*n* = 79,372	*n* = 142,679		*n* = 74,346	*n* = 73,603	*n* = 74,102	
Demographics							
Mean age, years	73.4 (12.7)	72.4 (13.5)	<0.001	73.7 (12.6)	72.4 (13.2)	72.3 (13.7)	<0.001
Female sex	49.2	50.6	<0.001	49.5	50.0	50.8	<0.001
Income quartiles			<0.001				<0.001
1^st^ (lowest)	31.6	20.1		32.6	22.7	17.3	
2^nd^	25.2	25.4		25.3	26.0	24.7	
3^rd^	23.3	26.5		23.1	26.6	26.4	
4^th^ (highest)	19.9	28.0		19.0	24.8	31.6	
Comorbidities							
Abnormal liver function	0.4	0.5	0.002	0.4	0.5	0.6	<0.001
Abnormal renal function	3.8	4.1	<0.001	3.8	3.9	4.3	<0.001
Alcohol use disorder	3.7	4.1	<0.001	3.8	3.9	4.3	<0.001
Any vascular disease	29.7	27.1	<0.001	29.8	28.4	25.8	<0.001
Cancer	19.7	21.3	<0.001	19.7	20.2	22.3	<0.001
Dementia	5.2	5.2	0.902	5.3	4.9	5.3	<0.001
Diabetes	22.5	21.2	<0.001	22.6	21.7	20.7	<0.001
Dyslipidemia	48.2	48.0	0.347	48.4	49.1	46.6	<0.001
Heart failure	18.2	16.9	<0.001	18.5	17.3	16.2	<0.001
Hypertension	75.2	73.9	<0.001	75.3	74.2	73.6	<0.001
Prior bleeding	10.6	10.9	0.103	10.7	10.9	10.7	0.450
Prior IS or TIA	16.0	15.2	<0.001	16.2	15.6	14.7	<0.001
Psychiatric disorder	12.8	14.2	<0.001	13.0	13.7	14.3	<0.001
Risk scores							
Modified HAS-BLED score	2.5 (1.0)	2.5 (1.1)	<0.001	2.5 (1.0)	2.5 (1.1)	2.5 (1.1)	<0.001
CHA_2_DS_2_-VASc score	3.5 (1.9)	3.4 (1.9)	<0.001	3.5 (1.9)	3.4 (1.9)	3.4 (1.9)	<0.001

*Note*: IS: ischemic stroke; TIA: transient ischemic attack.

Values denote *n* (%) or mean (standard deviation). CHA_2_DS_2_-VASc score: congestive heart failure (1 point), hypertension (1 point), age ⩾75 years (2 points), diabetes (1 point), history of stroke or TIA (2 points), vascular disease (1 point), age 65–74 years (1 point), sex category (female) (1 point); modified HAS-BLED score: hypertension (1 point), abnormal renal or liver function (1 point each), prior stroke (1 point), bleeding history (1 point), age >65 years (1 point), alcohol abuse (1 point), concomitant antiplatelet/NSAIDs (1 point) (no labile INR, max score 8). Low stroke risk: men with CHA_2_DS_2_-VASc score 0 and women with CHA_2_DS_2_-VASc score ⩽ 1; intermediate stroke risk: men with CHA_2_DS_2_-VASc score 1 and women with CHA_2_DS_2_-VASc score 2; high stroke risk: men with CHA_2_DS_2_-VASc score >1 and women with CHA_2_DS_2_-VASc score > 2.

**Figure 1.**
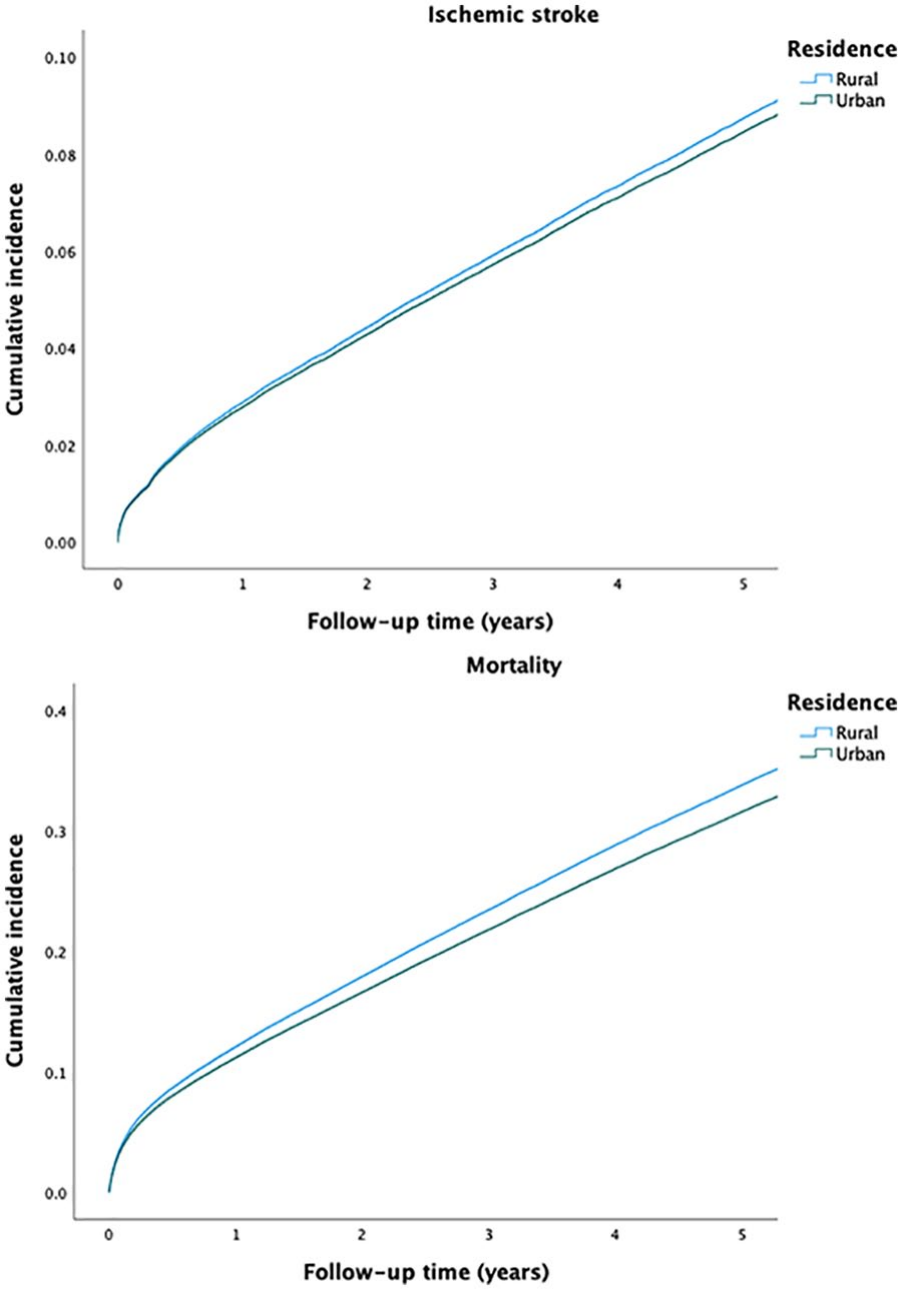
Crude incidence curves of ischemic stroke (above) and mortality (below) according to rural and urban residence.

The crude incidence of IS was similar in patients with rural and urban residence. However, the crude IS rate was lower in higher urbanization degree tertiles, when compared to the lowest. A lower crude mortality rate was observed in patients with urban residence or a higher urbanization degree tertile (Figure 1, Table II).

**Table II.** Incidence rates for ischemic stroke and death according to residence status.

	P-years (1000 years)	Events, *n*	Incidence (per 1000 P-years)	Unadjusted IRR	Adjusted IRR (Model 1)	Adjusted IRR (Model 2)	Adjusted IRR (Model 3)
Ischemic stroke							
Residence							
Rural	308.45	5652	18.3 (17.9–18.8)	Reference	Reference	Reference	Reference
Urban	560.59	9915	17.7 (17.3–18.0)	0.97 (0.93–1.00)	1.00 (0.97–1.03)	1.05 (1.01–1.08)	1.03 (1.00–1.07)
Urbanization degree tertile							
Lowest	286.10	5377	18.8 (18.3–19.3)	Reference	Reference	Reference	Reference
Middle	290.50	4991	17.2 (16.7–17.7)	0.91 (0.88–0.95)	0.96 (0.92–1.0)	0.99 (0.96–1.03)	0.99 (0.95–1.02)
Highest	292.45	5199	17.8 (17.3–18.3)	0.95 (0.91–0.98)	0.99 (0.94–1.03)	1.06 (1.02–1.10)	1.04 (1.00–1.08)
Mortality							
Residence							
Rural	321.9	27 182	84.4 (83.4–85.4)	Reference	Reference	Reference	Reference
Urban	584.7	45 383	77.6 (76.9–78.3)	0.92 (0.91–0.93)	0.97 (0.96–0.98)	1.06 (1.04–1.07)	1.02 (1.00–1.04)
Urbanization degree tertile							
Lowest	298.9	25 846	86.5 (85.4–87.5)	Reference	Reference	Reference	Reference
Middle	302.2	22 999	76.1 (75.1–77.1)	0.88 (0.86–0.90)	0.95 (0.93–0.96)	1.01 (0.99–1.03)	0.99 (0.97–1.01)
Highest	305.6	23 720	77.6 (76.6–78.6)	0.90 (0.88–0.91)	0.96 (0.95–0.98)	1.10 (1.08–1.12)	1.04 (1.02–1.06)

*Note*: IRR: incidence rate ratio; P-year: patient-year. IRRs estimated with Poisson regression. Model 1 adjusted for age, sex and calendar year. Model 2 further adjusted for baseline patient characteristics. Model 3 further adjusted for oral anticoagulant use during follow-up. 95% confidence intervals in parenthesis.

After adjusting for patient characteristics, urban residence was associated with a higher IS rate. The highest urbanization degree tertile was also associated with a higher IS rate when compared to the lowest (Table II). The findings were materially similar in patients under and over 65 years of age at AF diagnosis (Supplementary Table 2). Furthermore, the findings were echoed in the sensitivity anal-yses that also considered deaths caused by IS (Supplementary Table 3). When income and comorbidities were considered separately in the regression models, urban residence was associated with a higher IS rate after adjusting for income but not after adjusting for comorbidities (Supplementary Table 4). Correspondingly, urban residence and the highest urbanization degree tertile were associated with a higher mortality rate after controlling for differences in patient characteristics. When the regression models were further adjusted for OAC-use during follow-up, the rate ratios attenuated, but urban residence and the highest urbanization degree tertile remained associated with both a higher IS and mortality rate (Table II).

**Figure 2.**
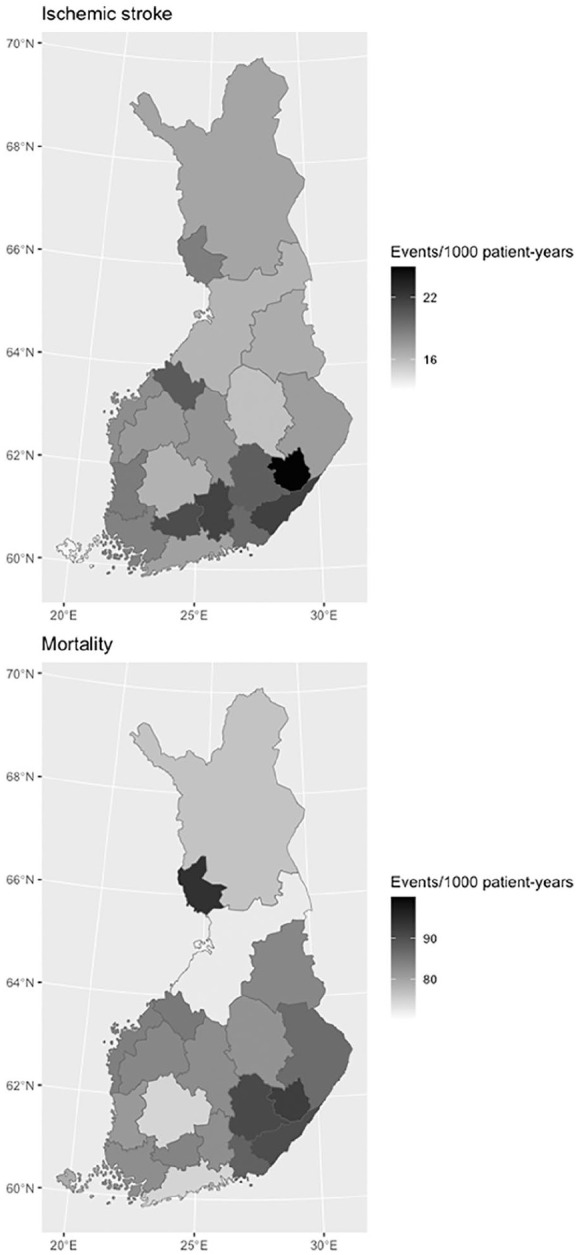
Crude ischemic stroke (above) and mortality (below) rates for hospital districts in Finland.

The outcome event rates varied between HDs (Figure 2). The highest crude IS rate was in the East Savo HD and the lowest in Åland. The highest crude mortality rate was in the Länsi-Pohja HD and the lowest in the North Ostrobothnia HD (Supplementary Table 5). After adjusting for age, calendar year, and sex, the highest IS and mortality rates were both in the East Savo HD, while the lowest IS rate was in Åland and the lowest mortality rates were in Åland and in the North Ostrobothnia HD (Supplementary Table 6).

## Discussion

This nationwide retrospective cohort study documented geographical differences in the prognosis of patients with incident AF in Finland between 2007 and 2018. Overall, the rural–urban disparities in IS and mortality rates appeared minimal, whereas considerable outcome disparities were observed between HDs.

Previous studies have reported higher all-cause mortality and the generally worse outcomes of cardiovascular diseases in rural populations, although some inconsistencies exist in their findings [5–11]. Regarding patients with AF, two Canadian studies have reported similar stroke and bleeding rates in patients with rural and urban residence [12, 13]. On the other hand, a study conducted in the United States observed higher mortality in patients hospitalized for AF in rural areas [14]. However, these studies have focused on selected patient populations and areas, which may limit the interpretation and generalizability of their findings. Considering the complete nationwide coverage of the study cohort, our results provide new and more robust data on rural–urban outcome differences in patients with AF.

The crude mortality rate was 8% lower in patients with urban residence, but the difference attenuated almost completely after adjusting for age, calendar year and sex (Model 1). After further adjusting for comorbidities, urban residence was associated with an approximately 5% higher rate of both IS and mortality (Model 2). Hence, the higher crude mortality in rural patients appeared to be related to their higher age and comorbidity. Differences in OAC-use seemed to explain part of the association between higher outcome rates and urban residence, since the rate ratios attenuated slightly after adjusting for OAC-use (Model 3). Taken together, rural–urban differences in IS and mortality rates seemed minimal and are unlikely to have clinical importance in the risk stratification of patients with AF. Furthermore, no meaningful inequality in the prognosis of patients with AF between rural and urban areas could be shown. These findings are concordant with the two previously published studies from the authors assessing the treatment of AF in Finland, which observed only a marginally lower use of OACs in patients with AF and urban residence and no meaningful differences in the use of rhythm control therapies, except for a slightly higher use of antiarrhythmic drugs in urban areas [20, 26]. Likewise, our findings are in concordance with the two Canadian studies reporting similar outcomes in AF patients with rural and urban residence [12, 13].

Compared with the rural–urban axis, the outcome differences between HDs were more striking. Even after controlling for differences in age structures, there was an almost twofold difference in the IS incidence rates between Eastern Savo and Åland. The mortality disparities were somewhat smaller, but still an approximately 20% difference was observed between the HDs with the highest and lowest age-adjusted mortality rates. Comorbidity and OAC-use seemed to partly, but not entirely, explain these outcome differences, since the rate ratios abated but still clearly differed after adjusting for these factors (Supplementary Table 4). Possible variability in AF diagnostics may have led to earlier detection of the arrhythmia in certain areas, which could also be reflected in the outcome differences. On the whole, our results concurred with previous observations of generally higher cardiovascular morbidity and mortality in eastern Finland, although some additional geographical granularity was also observed [16, 17, 27]. Genetic risk factors have been proposed to explain part of this east–west health variation [27–29].

The most important limitations of the current study are related to its retrospective registry-based study design. Hence, information bias may be present owing to inappropriately recorded or unmeasured data, and differences in health care utilization may have affected data on the prevalence of the comorbidities. Therefore, despite the comprehensive set of variables used in the adjustments, residual confounding cannot be excluded. Additionally, it is important to acknowledge that although our analyses considered several comorbidities, they did not provide insights into the precise influence of previously observed health trends, such as the decrease in alcohol use or the prevalence of ischemic heart disease, on the disparities in prognoses between rural and urban areas, highlighting the need for future studies to examine these aspects more comprehensively [30, 31]. Furthermore, our results represent associations, not necessarily causal relationships between patients’ residence and outcomes. The administrative data used lacked information about lifestyle-related factors, except for the diagnosed alcohol use disorders, and these factors presumably contribute to outcome rates. Despite these limitations, a particular strength of our study is the uniquely comprehensive nationwide coverage of the linked national registries. Indeed, the data cover all patients with AF from all levels of care in Finland, as well as all their OAC purchases and recorded outcome events. Moreover, the hospital care register used to define the IS outcome is well-validated and has relatively high diagnostic accuracy, especially regarding cardiovascular diseases [32].

In conclusion, this nationwide retrospective cohort study documented that urban residence was associated with a marginally higher IS and mortality rate in patients with AF in Finland. However, considering the small effect size, there appeared to be no clinically meaningful rural–urban inequality in the prognosis of AF. On the other hand, significant differences were found between HDs in IS and mortality rates. The factors underlying these differences should be explored in future studies to develop interventions to reduce disparities in the outcomes of patients with AF.

## Supplemental Material

sj-docx-1-sjp-10.1177_14034948231189918 – Supplemental material for Rural–urban and geographical differences in prognosis of atrial fibrillation in Finland: a nationwide cohort study Supplemental material, sj-docx-1-sjp-10.1177_14034948231189918 for Rural–urban and geographical differences in prognosis of atrial fibrillation in Finland: a nationwide cohort study by Konsta Teppo, K.E. Juhani Airaksinen, Olli Halminen, Jussi Jaakkola, Miika Linna, Jari Haukka, Jukka Putaala, Pirjo Mustonen, Janne Kinnunen, Juha Hartikainen and Mika Lehto in Scandinavian Journal of Public Health
