# Supplementary material for: Rural–urban and geographical differences in prognosis of atrial fibrillation in Finland: a nationwide cohort study
Source: Scand J Public Health. 2023 Aug 12;52(7):785–92. doi: 10.1177/14034948231189918 (PMC11487984; doi:10.1177/14034948231189918)

**Supplementary Material**

**Supplementary Table 1.** Definitions of the comorbidities

**Supplementary Table 2.** Incidence rates of ischemic stroke according to rural and urban residence in patients under and over 65 years of age at the time of atrial fibrillation diagnosis

**Supplementary Table 3.** Incidence rates of ischemic stroke according to rural and urban residence in the sensitivity analyses including also deaths caused by ischemic stroke

**Supplementary Table 4.** Incidence rates of ischemic stroke according to rural and urban residence in the sensitivity analyses adjusted for comorbidities and income separately

**Supplementary Table 5.** Crude incidence rates for ischemic stroke and death according to hospital districts

**Supplementary Table 6.** Adjusted incidence rate ratios for ischemic stroke and death according to hospital districts

**Supplementary Table 7.** Mean age and risk scores according to hospital districts

**Supplementary Figure 1.** Flow-chart of the patient selection process

**Supplementary Table 1**. Definitions of the comorbidities

|  | ICD-10 | ICPC-2 | Reimbursement code | ATC code | Other |
| --- | --- | --- | --- | --- | --- |
| Any vascular disease | I20-I25, I65-I66, I67.2, I70 | K74, K75, K76, K91, K92 | 206 |  |  |
| Hypertension | I10-I15 | K85, K86, K87 | 205 | C03A, C03B, C03DB, C03EA, C07A, C08CA, C08D, C09 |  |
| Dyslipidemia | E78 | T93 | 206 | C10 |  |
| Heart failure | I50, I11.0, I13.0, I13.2 | K77 | 201 |  |  |
| Diabetes | E10-E14 | T89, T90 | 103, 215 | A10 |  |
| Previous stroke | I63, I64, I69.3-I69.8 | K90 |  |  |  |
| Bleeding history | D50.0, D62, D68.3, I60-I62, I69.0-I69.2, I85.0, I86.4, J94.2, K22.1, K22.3, K22.6, K25.0, K25.2, K25.4, K25.6, K26.0, K26.2, K26.4, K26.6, K27.0, K27.2, K27.4, K27.6, K28.0, K28.2, K28.4, K28.6, K29.0, K62.5, K63.1, K63.3, K92.0-K92.2, N02, R04, R31, R58, S06.2-S06.6, S06.8 |  |  |  |  |
| Alcohol abuse | F10 |  |  |  |  |
| Renal failure or dialysis | N18, Z49 |  |  |  |  |
| Liver cirrhosis or failure | K70.2-K70.4, K71.7, K71.8, K72, K74 |  |  |  |  |
| Dementia | F00-F03, G30 |  |  |  |  |
| Cancer |  |  |  |  | Any cancer registered in the Finnish Cancer Registry |
| Psychiatric disorder | F04-F99 |  |  |  |  |

Abbreviations: ATC, anatomic therapeutic chemical; ICD-10, International Classification of Diseases, Tenth Revision; ICPC-2, International Classification of Primary Care, Second Editio

**Supplementary Table 2.** Incidence rates of ischemic stroke according to rural and urban residence in patients under and over 65 years of age at the time of atrial fibrillation diagnosis

|  | | **P-years (1000 years)** | | **Events, n** | | **Incidence (per 1000 p-years)** | | **Unadjusted IRR** | **Adjusted IRR (Model 1)** | | **Adjusted IRR (Model 2)** | | **Adjusted IRR (Model 3)** | |
| --- | --- | --- | --- | --- | --- | --- | --- | --- | --- | --- | --- | --- | --- | --- |
| **Under 65 years** | |  | |  | |  |  | |  | |  | |  | |
| Rural | | 96.26 | | 730 | | 7.58 (7.04-8.15) | (Reference) | | (Reference) | | (Reference) | | (Reference) | |
| Urban | | 189.41 | | 1 396 | | 7.37 (6.99-7.77) | 0.97 (0.89-1.06) | | 1.00 (0.91-1.09) | | 1.05 (0.96-1.15) | | 1.05 (0.96-1.15) | |
| **65 years or more** | |  | |  | |  | |  |  | |  | |  | |
| Rural | | 212.20 | | 4 922 | | 23.20 (22.55-23.85) | | (Reference) | (Reference) | | (Reference) | | (Reference) | |
| Urban | | 371.18 | | 8 519 | | 22.95 (22.47-23.44) | | 0.99 (0.96-1.02) | 1.01 (0.97-1.04) | | 1.05 (1.01-1.08) | | 1.02 (0.99-1.06) | |
| Abbreviations: IRR, incidence rate ratio; P-year, patient-year. IRRs estimated with the Poisson regression. Model 1 adjusted for age, sex and calendar year. Model 2 further adjusted for baseline patient characteristics. Model 3 further adjusted for oral anticoagulant use during follow-up. 95% confidence intervals in parenthesis. | | | | | | | | | | | | | | |

**Supplementary Table 3.** Incidence rates of ischemic stroke according to rural and urban residence in the sensitivity analyses including also deaths caused by ischemic stroke

|  | **P-years (1000 years)** | **Events, n** | **Incidence (per 1000 p-years)** | **Unadjusted IRR** | **Adjusted IRR (Model 1)** | **Adjusted IRR (Model 2)** | **Adjusted IRR (Model 3)** |
| --- | --- | --- | --- | --- | --- | --- | --- |
| **Ischemic stroke** |  |  |  |  |  |  |  |
| **Residence** |  |  |  |  |  |  |  |
| Rural | 308.45 | 6 391 | 20.7 (20.2-21.2) | (Reference) | (Reference) | (Reference) | (Reference) |
| Urban | 560.59 | 11 072 | 19.8 (19.4-20.1) | 0.95 (0.92-0.98) | 0.99 (0.96-1.02) | 1.04 (1.01-1.08) | 1.02 (0.99-1.05) |
| Abbreviations: IRR, incidence rate ratio; P-year, patient-year. IRRs estimated with the Poisson regression. Model 1 adjusted for age, sex and calendar year. Model 2 further adjusted for baseline patient characteristics. Model 3 further adjusted for oral anticoagulant use during follow-up. 95% confidence intervals in parenthesis. | | | | | | | |

**Supplementary Table 4.** Incidence rates of ischemic stroke according to rural and urban residence in the sensitivity analyses adjusted for comorbidities and income separately

|  | | **Unadjusted IRR** | | **IRRs adjusted for age, sex, calendar year and comorbidities** | | **IRRs adjusted for age, sex, calendar year and income** |
| --- | --- | --- | --- | --- | --- | --- |
| Rural | | (Reference) | | (Reference) | (Reference) |  |
| Urban | | 0.97 (0.93-1.00) | | 1.01 (0.97-1.04) | 1.06 (1.02-1.09) |  |
| Abbreviations: IRR, incidence rate ratio. IRRs estimated with the Poisson regression. | | | | | | |

**Supplementary Table 5.** Crude incidence rates for ischemic stroke and death according to hospital districts

| **Hospital district** | **P-years (1000 years)** | **Events, n** | **Incidence rate (per 1000 p-years)** | **Incidence rate 95% CI lower limit** | **Incidence rate 95% CI upper limit** | **Unadjusted IRR** | **IRR 95% CI lower limit** | **IRR 95% CI upper limit** |
| --- | --- | --- | --- | --- | --- | --- | --- | --- |
| **Ischemic stroke** | | |  |  |  |  |  |  |
| Helsinki and Uusimaa | 200.87 | 3424 | 17.05 | 16.48 | 17.63 | (ref) | (ref) | (ref) |
| Southwest Finland | 80.44 | 1487 | 18.49 | 17.56 | 19.45 | 1.08 | 1.02 | 1.15 |
| Satakunta | 43.68 | 821 | 18.79 | 17.53 | 20.13 | 1.10 | 1.02 | 1.19 |
| Kanta-Häme | 30.76 | 648 | 21.06 | 19.47 | 22.75 | 1.24 | 1.14 | 1.34 |
| Pirkanmaa | 95.04 | 1548 | 16.29 | 15.49 | 17.12 | 0.96 | 0.90 | 1.01 |
| Päijät-Häme | 35.63 | 771 | 21.64 | 20.14 | 23.22 | 1.27 | 1.17 | 1.37 |
| Kymenlaakso | 31.94 | 627 | 19.63 | 18.12 | 21.23 | 1.15 | 1.06 | 1.25 |
| South Karelia | 26.76 | 582 | 21.75 | 20.02 | 23.59 | 1.28 | 1.17 | 1.39 |
| South Savo | 23.11 | 467 | 20.21 | 18.42 | 22.13 | 1.19 | 1.08 | 1.31 |
| East Savo | 9.33 | 232 | 24.87 | 21.77 | 28.28 | 1.46 | 1.28 | 1.67 |
| North Karelia | 32.42 | 558 | 17.21 | 15.81 | 18.70 | 1.01 | 0.92 | 1.10 |
| North Savo | 44.96 | 705 | 15.68 | 14.55 | 16.88 | 0.92 | 0.85 | 1.00 |
| Central Finland | 37.58 | 662 | 17.62 | 16.30 | 19.01 | 1.03 | 0.95 | 1.12 |
| South Ostrobothnia | 33.42 | 582 | 17.42 | 16.03 | 18.89 | 1.02 | 0.94 | 1.12 |
| Vaasa | 25.69 | 461 | 17.94 | 16.34 | 19.66 | 1.05 | 0.96 | 1.16 |
| Central Ostrobothnia | 13.52 | 275 | 20.34 | 18.00 | 22.89 | 1.19 | 1.06 | 1.35 |
| North Ostrobothnia | 51.97 | 843 | 16.22 | 15.14 | 17.35 | 0.95 | 0.88 | 1.03 |
| Kainuu | 15.04 | 249 | 16.56 | 14.57 | 18.75 | 0.97 | 0.85 | 1.10 |
| Länsi-Pohja | 10.87 | 203 | 18.67 | 16.19 | 21.42 | 1.10 | 0.95 | 1.26 |
| Lapland | 20.90 | 353 | 16.89 | 15.18 | 18.75 | 0.99 | 0.89 | 1.11 |
| Åland | 5.11 | 69 | 13.51 | 10.51 | 17.09 | 0.79 | 0.62 | 1.01 |
| **Mortality** |  |  |  |  |  |  |  |  |
| Helsinki and Uusimaa | 209.36 | 15612 | 74.57 | 73.40 | 75.75 | (ref) | (ref) | (ref) |
| Southwest Finland | 83.99 | 6915 | 82.33 | 80.40 | 84.29 | 1.10 | 1.07 | 1.14 |
| Satakunta | 45.74 | 3707 | 81.05 | 78.46 | 83.70 | 1.09 | 1.05 | 1.13 |
| Kanta-Häme | 32.31 | 2695 | 83.41 | 80.29 | 86.62 | 1.12 | 1.07 | 1.17 |
| Pirkanmaa | 98.80 | 7350 | 74.40 | 72.70 | 76.12 | 1.00 | 0.97 | 1.03 |
| Päijät-Häme | 37.38 | 3071 | 82.16 | 79.28 | 85.12 | 1.10 | 1.06 | 1.15 |
| Kymenlaakso | 33.40 | 2923 | 87.51 | 84.37 | 90.75 | 1.17 | 1.13 | 1.22 |
| South Karelia | 28.26 | 2548 | 90.16 | 86.69 | 93.73 | 1.21 | 1.16 | 1.26 |
| South Savo | 24.15 | 2189 | 90.66 | 86.90 | 94.54 | 1.22 | 1.16 | 1.27 |
| East Savo | 9.79 | 902 | 92.12 | 86.20 | 98.33 | 1.24 | 1.16 | 1.32 |
| North Karelia | 33.78 | 2915 | 86.29 | 83.19 | 89.48 | 1.16 | 1.11 | 1.20 |
| North Savo | 46.64 | 3817 | 81.84 | 79.26 | 84.47 | 1.10 | 1.06 | 1.14 |
| Central Finland | 39.19 | 3230 | 82.43 | 79.61 | 85.32 | 1.11 | 1.06 | 1.15 |
| South Ostrobothnia | 34.81 | 2893 | 83.12 | 80.12 | 86.20 | 1.11 | 1.07 | 1.16 |
| Vaasa | 26.80 | 2251 | 84.00 | 80.57 | 87.54 | 1.13 | 1.08 | 1.18 |
| Central Ostrobothnia | 14.18 | 1202 | 84.75 | 80.02 | 89.68 | 1.14 | 1.07 | 1.21 |
| North Ostrobothnia | 54.13 | 3906 | 72.16 | 69.91 | 74.46 | 0.97 | 0.93 | 1.00 |
| Kainuu | 15.61 | 1298 | 83.16 | 78.70 | 87.81 | 1.12 | 1.05 | 1.18 |
| Länsi-Pohja | 11.32 | 1063 | 93.86 | 88.31 | 99.68 | 1.26 | 1.18 | 1.34 |
| Lapland | 21.79 | 1665 | 76.41 | 72.78 | 80.17 | 1.02 | 0.97 | 1.08 |
| Åland | 5.23 | 413 | 78.95 | 71.52 | 86.95 | 1.06 | 0.96 | 1.17 |
| Abbreviations: IRR, incidence rate ratio; P-year, patient-year. IRRs estimated with the Poisson regression. | | | | | | | | |

**Supplementary Table 6.** Adjusted incidence rate ratios for ischemic stroke and death according to hospital districts

| **Hospital district** | **Model 1 Adjusted IRR** | **Model 1 IRR 95% CI lower limit** | **Model 1 IRR 95% CI upper limit** | **Model 2**  **Adjusted IRR** | **Model 2 IRR 95% CI lower limit** | **Model 2 IRR 95% CI upper limit** | **Model 3**  **Adjusted IRR** | **Model 3 IRR 95% CI lower limit** | **Model 3 IRR 95% CI upper limit** |
| --- | --- | --- | --- | --- | --- | --- | --- | --- | --- |
| **Ischemic stroke** | | |  |  |  |  |  |  |  |
| Helsinki and Uusimaa | (ref) | (ref) | (ref) | (ref) | (ref) | (ref) | (ref) | (ref) | (ref) |
| Southwest Finland | 1.01 | 0.95 | 1.07 | 0.96 | 0.90 | 1.02 | 0.96 | 0.90 | 1.02 |
| Satakunta | 1.04 | 0.96 | 1.12 | 0.98 | 0.91 | 1.06 | 1.02 | 0.94 | 1.10 |
| Kanta-Häme | 1.17 | 1.08 | 1.27 | 1.11 | 1.02 | 1.21 | 1.08 | 0.99 | 1.17 |
| Pirkanmaa | 0.94 | 0.89 | 1.00 | 0.90 | 0.85 | 0.95 | 0.90 | 0.85 | 0.96 |
| Päijät-Häme | 1.23 | 1.13 | 1.33 | 1.16 | 1.08 | 1.26 | 1.15 | 1.06 | 1.24 |
| Kymenlaakso | 1.09 | 1.00 | 1.19 | 1.03 | 0.95 | 1.13 | 1.01 | 0.93 | 1.10 |
| South Karelia | 1.17 | 1.07 | 1.28 | 1.12 | 1.02 | 1.22 | 1.13 | 1.04 | 1.24 |
| South Savo | 1.10 | 1.00 | 1.21 | 1.02 | 0.93 | 1.12 | 1.07 | 0.97 | 1.18 |
| East Savo | 1.35 | 1.18 | 1.54 | 1.23 | 1.07 | 1.40 | 1.24 | 1.09 | 1.42 |
| North Karelia | 0.95 | 0.87 | 1.04 | 0.87 | 0.80 | 0.95 | 0.91 | 0.83 | 0.99 |
| North Savo | 0.87 | 0.80 | 0.94 | 0.80 | 0.74 | 0.87 | 0.83 | 0.76 | 0.90 |
| Central Finland | 0.99 | 0.91 | 1.08 | 0.93 | 0.86 | 1.01 | 0.98 | 0.90 | 1.06 |
| South Ostrobothnia | 0.94 | 0.86 | 1.03 | 0.86 | 0.79 | 0.94 | 0.91 | 0.83 | 0.99 |
| Vaasa | 0.96 | 0.87 | 1.05 | 0.92 | 0.83 | 1.01 | 0.92 | 0.84 | 1.02 |
| Central Ostrobothnia | 1.10 | 0.97 | 1.25 | 1.01 | 0.89 | 1.14 | 1.04 | 0.92 | 1.17 |
| North Ostrobothnia | 0.93 | 0.86 | 1.00 | 0.87 | 0.80 | 0.93 | 0.87 | 0.81 | 0.94 |
| Kainuu | 0.95 | 0.84 | 1.08 | 0.88 | 0.77 | 1.00 | 0.87 | 0.77 | 0.99 |
| Länsi-Pohja | 0.99 | 0.86 | 1.14 | 0.92 | 0.80 | 1.07 | 0.95 | 0.82 | 1.09 |
| Lapland | 0.97 | 0.87 | 1.08 | 0.92 | 0.82 | 1.02 | 0.92 | 0.83 | 1.03 |
| Åland | 0.75 | 0.59 | 0.95 | 0.78 | 0.61 | 0.99 | 0.76 | 0.60 | 0.97 |
| **Mortality** |  |  |  |  |  |  |  |  |  |
| Helsinki and Uusimaa | (ref) | (ref) | (ref) | (ref) | (ref) | (ref) | (ref) | (ref) | (ref) |
| Southwest Finland | 0.97 | 0.94 | 1.00 | 0.87 | 0.84 | 0.89 | 0.88 | 0.86 | 0.91 |
| Satakunta | 1.00 | 0.97 | 1.04 | 0.93 | 0.89 | 0.96 | 0.99 | 0.96 | 1.03 |
| Kanta-Häme | 1.02 | 0.98 | 1.07 | 0.92 | 0.88 | 0.96 | 0.88 | 0.85 | 0.92 |
| Pirkanmaa | 0.97 | 0.94 | 0.99 | 0.86 | 0.84 | 0.89 | 0.88 | 0.86 | 0.91 |
| Päijät-Häme | 1.05 | 1.01 | 1.09 | 0.95 | 0.91 | 0.98 | 0.94 | 0.91 | 0.98 |
| Kymenlaakso | 1.08 | 1.03 | 1.12 | 1.01 | 0.97 | 1.05 | 1.01 | 0.97 | 1.05 |
| South Karelia | 1.07 | 1.03 | 1.12 | 0.97 | 0.93 | 1.02 | 1.01 | 0.97 | 1.05 |
| South Savo | 1.10 | 1.05 | 1.15 | 0.95 | 0.91 | 1.00 | 1.06 | 1.01 | 1.11 |
| East Savo | 1.12 | 1.05 | 1.20 | 0.94 | 0.88 | 1.00 | 0.97 | 0.91 | 1.04 |
| North Karelia | 1.08 | 1.04 | 1.12 | 0.92 | 0.88 | 0.96 | 1.00 | 0.96 | 1.04 |
| North Savo | 1.02 | 0.98 | 1.05 | 0.87 | 0.84 | 0.90 | 0.92 | 0.89 | 0.95 |
| Central Finland | 1.04 | 1.00 | 1.08 | 0.93 | 0.89 | 0.97 | 1.03 | 1.00 | 1.07 |
| South Ostrobothnia | 0.97 | 0.93 | 1.00 | 0.78 | 0.75 | 0.81 | 0.88 | 0.84 | 0.91 |
| Vaasa | 0.96 | 0.92 | 1.00 | 0.87 | 0.83 | 0.91 | 0.88 | 0.84 | 0.92 |
| Central Ostrobothnia | 1.02 | 0.96 | 1.08 | 0.90 | 0.85 | 0.95 | 0.96 | 0.91 | 1.02 |
| North Ostrobothnia | 0.94 | 0.91 | 0.97 | 0.85 | 0.82 | 0.88 | 0.89 | 0.86 | 0.92 |
| Kainuu | 1.09 | 1.03 | 1.16 | 0.95 | 0.90 | 1.01 | 0.98 | 0.93 | 1.04 |
| Länsi-Pohja | 1.07 | 1.00 | 1.13 | 0.93 | 0.87 | 0.98 | 0.96 | 0.90 | 1.02 |
| Lapland | 1.01 | 0.96 | 1.06 | 0.91 | 0.87 | 0.96 | 0.96 | 0.91 | 1.01 |
| Åland | 0.94 | 0.85 | 1.03 | 0.96 | 0.87 | 1.06 | 0.88 | 0.80 | 0.97 |
| Abbreviations: CI, confidence interval; IRR, incidence rate ratio. IRRs estimated with the Poisson regression. Model 1 adjusted for age, sex and calendar year. Model 2 further adjusted for baseline patient characteristics. Model 3 further adjusted for oral anticoagulant use during follow-up. | | | | | | | | | |

**Supplementary Table 7.** Mean age and risk scores according to hospital districts

| **Hospital district** | **Age** | **CHA_2_DS_2_-VASc score** | **Modified HAS-BLED score** |
| --- | --- | --- | --- |
| Helsinki and Uusimaa | 71.6 (13.6) | 3.3 (1.9) | 2.5 (1.1) |
| Southwest Finland | 73.6 (13.1) | 3.5 (1.9) | 2.6 (1.1) |
| Satakunta | 73.1 (12.7) | 3.5 (1.8) | 2.5 (1.0) |
| Kanta-Häme | 73.3 (13.2) | 3.5 (1.9) | 2.5 (1.1) |
| Pirkanmaa | 72.2 (13.8) | 3.4 (1.9) | 2.5 (1.1) |
| Päijät-Häme | 73.1 (12.8) | 3.5 (1.9) | 2.6 (1.1) |
| Kymenlaakso | 73.5 (12.6) | 3.5 (1.9) | 2.6 (1.0) |
| South Karelia | 74.1 (12.4) | 3.6 (1.9) | 2.5 (1.1) |
| South Savo | 73.9 (12.2) | 3.6 (1.9) | 2.5 (1.0) |
| East Savo | 74.3 (12.1) | 3.7 (2.0) | 2.6 (1.0) |
| North Karelia | 73.3 (12.5) | 3.6 (1.9) | 2.5 (1.0) |
| North Savo | 72.8 (13.2) | 3.5 (1.9) | 2.6 (1.0) |
| Central Finland | 72.9 (13.2) | 3.5 (1.9) | 2.5 (1.0) |
| South Ostrobothnia | 73.7 (13.1) | 3.3 (1.9) | 2.5 (1.0) |
| Vaasa | 74.1 (13.3) | 3.4 (1.9) | 2.4 (1.1) |
| Central Ostrobothnia | 73.6 (12.9) | 3.7 (1.9) | 2.6 (1.0) |
| North Ostrobothnia | 72.0 (13.4) | 3.3 (1.9) | 2.5 (1.1) |
| Kainuu | 72.8 (12.8) | 3.5 (1.9) | 2.5 (1.1) |
| Länsi-Pohja | 74.0 (12.7) | 3.7 (1.9) | 2.5 (1.0) |
| Lapland | 71.7 (13.2) | 3.3 (1.9) | 2.4 (1.0) |
| Åland | 73.6 (12.8) | 3.1 (1.7) | 2.3 (1.0) |
| Values denote mean (standard deviation). Abbreviations: CHA_2_DS_2_-VASc, congestive heart failure, hypertension, age ≥75 years, diabetes, history of stroke or TIA, vascular disease, age 65-74 years, sex category (female); modified HAS-BLED score, hypertension, abnormal renal or liver function, prior stroke, bleeding history, age >65 years, alcohol abuse, concomitant antiplatelet/NSAIDs (no labile INR, max score 8). P-value for difference in each variable between hospital districts <0.001. | | | |

**Supplementary Figure 1.** Flow-chart of the patient selection process


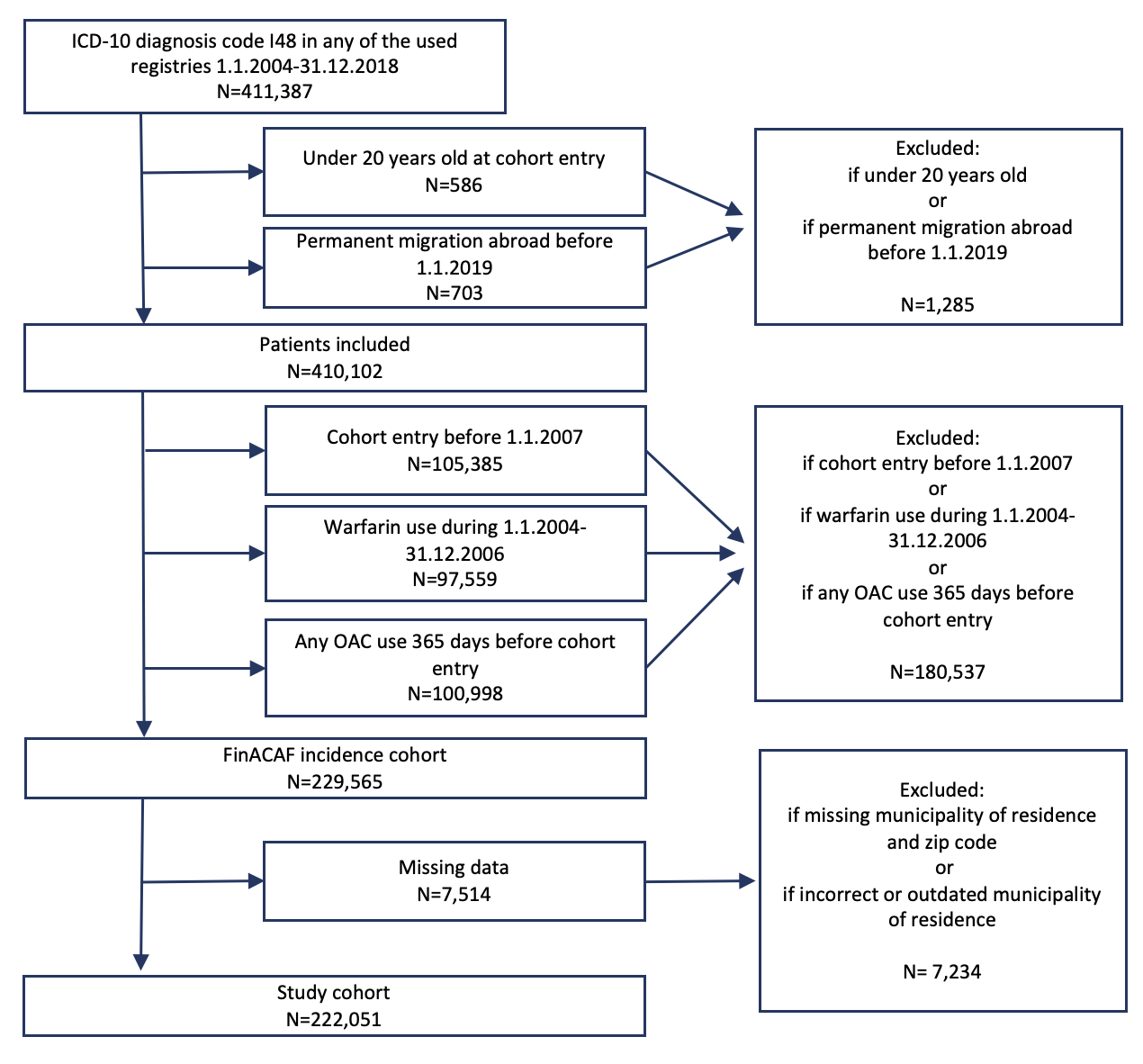

Supplement: sj-docx-1-sjp-10.1177_14034948231189918 – Supplemental material for Rural–urban and geographical differences in prognosis of atrial fibrillation in Finland: a nationwide cohort study [file sj-docx-1-sjp-10.1177_14034948231189918.docx]
